# Does urban–rural integration contribute to environmental health? Exploring the interplay between urban–rural integration and air quality dynamics in Yangtze River middle reaches city cluster

**DOI:** 10.3389/fpubh.2024.1496989

**Published:** 2025-01-07

**Authors:** Jixin Yang, Bowen Fu, Xufeng Cui

**Affiliations:** ^1^School of Business Administration, Zhongnan University of Economics and Law, Wuhan, China; ^2^Center for Land Economics, Zhongnan University of Economics and Law, Wuhan, China

**Keywords:** urban–rural integration, air quality, coupling coordination, geographical detector, Yangtze River middle reaches city cluster

## Abstract

**Background:**

Exploring the coordinated relationship between urban–rural integration and air quality has significant implications for promoting urban–rural development, preventing air pollution and ensuring residents’ health. This study takes Yangtze River middle reaches city cluster as a case study, calculates the levels of urban–rural integration and air quality development, analyzes their coupled coordination relationship and driving factors, and explores the path of coordinated development.

**Methods:**

This study constructs a coupling coordination degree model to analyze the relationship between the urban–rural integration development level and air quality development level. We use the trend surface method to analyze the spatial divergence characteristics of the coordination degree between urban–rural integration and air quality. In addition, we used a geographic detector to analyze the factors affecting the coordination degree.

**Results:**

(1) The overall level of urban–rural integration development showed an upward trend. High-value regions were concentrated in the Wuhan, Chang-Zhu-Tan, and Nanchang metropolitan areas. (2) The Air Quality Index showed an overall decline, with the most significant improvements observed in Wuhan, Changsha, and Jiujiang. (3) The coupling degree increased from 0.570 in 2013 to 0.794 in 2021, and the coordination degree increased from 0.337 in 2013 to 0.591 in 2021. The link between urban–rural integration and air quality has deepened over time, and the two promote each other, making city cluster develop towards environmental friendliness. The spatial distribution of coordination degree shows a “high in the west and low in the east, high in the north and low in the south” trend. (4) Per capita GDP, non-agricultural employment ratio, urban–rural spatial circulation media, population urbanization level, and fixed asset investment were identified as the core factors driving the coordination degree between urban–rural integration and air quality.

**Conclusion:**

This study confirms that the urban–rural integration and air quality of Yangtze River middle reaches city cluster are gradually changing in the direction of high-quality coordination. However, there are great differences among cities, regional imbalance is prominent, and coordination degree is driven by multidimensional factors.

## 1 Introduction

Air quality is a long-standing and complex issue that accompanies urbanization and has received extensive attention from scholars from diverse fields such as ecology, public health and urban development (1–3). It is worth noting that the process of urbanization has not been uniform across the globe, and in particular there are marked differences between developed and developing countries. Cities in developed countries, such as the United States of America and European countries, recognized the dangers of air pollution during the process of rapid urbanization and gradually restored air quality in their cities through the design and implementation of regulations, ultimately achieving a high level of urbanization (4). In contrast, developing countries are experiencing or have just begun to urbanize, and traditional patterns of urban development are generating large quantities of pollutants that will threaten air quality and public health (3, 5), for which sustainable development strategies are essential. As the largest developing country, China’s urbanization has entered the development stage of “urban–rural integration” (6), and a series of policies have been issued as support, including the *Opinions of the CPC Central Committee and The State Council on Establishing and Improving Institutions, Mechanisms and Policy Systems for Integrated Urban and Rural Development (2019)*, and *Government Work Report (2024)*. These measures have established a sustainable “urban–rural integration” mechanism and promoted the coordinated development of large, medium-sized and small cities, small towns and new rural communities. However, despite the significant achievements of urban–rural integration in China in terms of economic development (7), production (8), and social services (9), insufficient attention has been paid to the ecological environment. The large-scale construction of towns and villages poses challenges to the ecological environment, with air pollution being particularly prominent and exerting adverse effects on the health of residents (10–12). In the face of this phenomenon, the Chinese government has stressed the need to improve air quality in the process of development and has formulated the *Action Plan for Continuous Improvement of Air Quality (2023)*, which places higher requirements on the urban–rural atmospheric environment. Therefore, strengthening green, ecological and livable new urbanization and promoting urban–rural integration and air quality is an inevitable trend for China’s future high-quality development, which needs to be paid attention to by researchers.

Existing literature predominantly segregates research on urban–rural integration and air quality, with extensive scholarly discussions on each topic (13–16). Regarding urban–rural integration, existing research has primarily focused on measuring the level of integration (17, 18), identifying integration types (19), exploring influencing factors (20, 21), and proposing strategies to promote integration (22, 23). Among these, the interaction between urban–rural integration and influencing factors is of particular interest, with scholars examining the relationship between factors such as labor productivity, land use, poverty, topography, and urban–rural integration (24–26). The results indicate that urban–rural integration is significant in narrowing the urban–rural economic gap, promoting the rational allocation of urban–rural resources, and optimizing the spatial layout between urban and rural regions (8, 27). However, in terms of research focus, scholars favor the interaction between economic development, social governance, spatial changes, and urban–rural integration, with relatively few studies addressing the relationship between urban–rural integration and environmental protection. Despite China’s emphasis on green environmental protection and humanistic ideals (28, 29), research on the coordinated ecological development of urban–rural regions and the ecological well-being of urban–rural residents remains insufficient. Although some scholars recognize the importance of environmental protection under urban–rural integration (13, 30, 31), research directly linking urban–rural integration with air quality is scarce. Some studies have focused on the impact of urban–rural income disparity on changes in air quality (12, 32); however, they have only touched on the economic dimension of urban–rural integration. The relationship between urban–rural integration and air quality requires further exploration.

Regarding air quality, existing literature has explored the relationship between air quality and urbanization, economic growth, industrial policies, and public transportation, among other factors (28, 33, 34). For example, based on data from 282 cities in China, Lin and Zhu found that air quality weakened and then strengthened as urbanization progressed (35). Shi et al. found that the migration of populations from rural to urban regions contributes to increased emissions of air pollutants (31). Based on China’s subway operations, Xie et al. proposed that public transportation investment can reduce air pollution (36). These studies provide guidance for analyzing the relationship between air quality and urban–rural integration. However, the existing research has primarily explored the one-way impact of individual factors on air quality (3, 36, 37). There may be a bidirectional effect between urban–rural integration and air quality, i.e., air quality also affects the process of urban–rural integration, and a more in-depth study of the relationship is warranted. The environmental Kuznets theory provides insights to analyze the relationship between urban–rural integration and air quality (38, 39). Often, the environmental Kuznets curve is used to explain the relationship between economic development and the ecological environment, arguing that there is an inverted U-shaped relationship between the two. However, the curve also takes an N-shaped, monotonically increasing or decreasing, or even irregular shape due to differences in the external environment and different stages of development. Some scholars have identified the inflection point between urbanization and air pollution with the help of the environmental Kuznets theory (1). At the same time, from the perspective of systems theory, both urban–rural integration and air quality are constituent elements under the umbrella of sustainable urban–rural development, which can be viewed as two interacting systems (40). Through the analysis of environmental Kuznets theory and system theory, urban–rural integration and air quality have mutual feedback mechanism, and the interaction between them needs to be analyzed. In terms of research methodology, coupling coordination degree model is commonly used in research methods to analyze the coupling coordination relationship between two systems (41–44), widely applied in fields such as environment, economy, and society. Some scholars have introduced a coupling coordination degree model to analyze the coupling coordination relationship between economic development and air quality (38), which provides a methodological reference for this study.

In summary, existing research lacks direct attention to the connection between urban–rural integration and air quality. Promoting the coordinated development of urban–rural integration and air quality has become an urgent issue. This study employs a coupling coordination degree model to investigate the coupling coordination relationship between urban–rural integration and air quality. In terms of research region, city cluster are vital regions for advancing urban–rural integration in China’s new era and constitute the organic components of urban–rural systems (42). *The 14th Five-Year Plan for the Development of Yangtze River middle reaches city cluster* calls to accelerate the coordinated development of city cluster, advance air pollution control, and eliminate severe weather pollution, ultimately achieving the dual goals of coordinated regional development and improvement in ecological environment quality. Therefore, this study takes Yangtze River middle reaches city cluster as a case study, calculates the levels of urban–rural integration and air quality development, analyzes their coupled coordination relationship and driving factors, and explores the path of coordinated development. This study innovatively incorporates urban–rural integration and air quality into the same analytical framework, enriching the research on urban–rural integration at multiple spatial scales. These results provide a basis for formulating regionally coordinated development policies and serve as a reference for formulating policies to enhance air quality, thus having significant practical implications.

## 2 Theoretical framework

Unlike in the past, when population and resources were overly concentrated in large cities, China’s new urbanization emphasizes the transformation of the traditional development model and the coordinated development of large, medium and small cities, small towns and new rural communities through urban–rural integration (6). However, there are fewer existing studies that explore urban–rural integration and its ecological and green characteristics. In the context of heightened attention to air quality and public health, a critical question arises: how does urban–rural integration affect air quality? Given the lack of research on the relationship between urban–rural integration and air quality in the existing literature, this study primarily draws on insights from the existing body of work on urbanization and air quality.

Currently, numerous empirical studies by various scholars have examined the relationship between urbanization and air quality (41, 45), focusing on four main aspects. First, some argue that while urbanization promotes socio-economic development, it often comes at the expense of the environment, exacerbating air pollution (46, 47). Second, others contend that urbanization can reduce air pollution by enhancing the efficiency of resource use through the agglomeration of production factors in urban areas, thus lowering emissions (48, 49). Third, there is evidence suggesting a nonlinear relationship between urbanization and air quality. Although urbanization can lead to emission reductions, the effects of increased population density and pollution diffusion associated with urban expansion may also contribute to pollution dispersion (50, 51). Fourth, some researchers propose an interactive relationship between urbanization and air quality, characterized primarily by a coupling relationship (41, 52). Additionally, a limited number of studies have explored the connections between urban–rural integration and ecological environment (53), land use (54), and landscape patterns (55), but none have directly addressed air quality issues. Building on this foundation, the present study primarily adopts the framework of urbanization and air quality interactions, employing a coupling coordination degree model to investigate the mutual influence between urban–rural integration and air quality.

**Figure 1:**
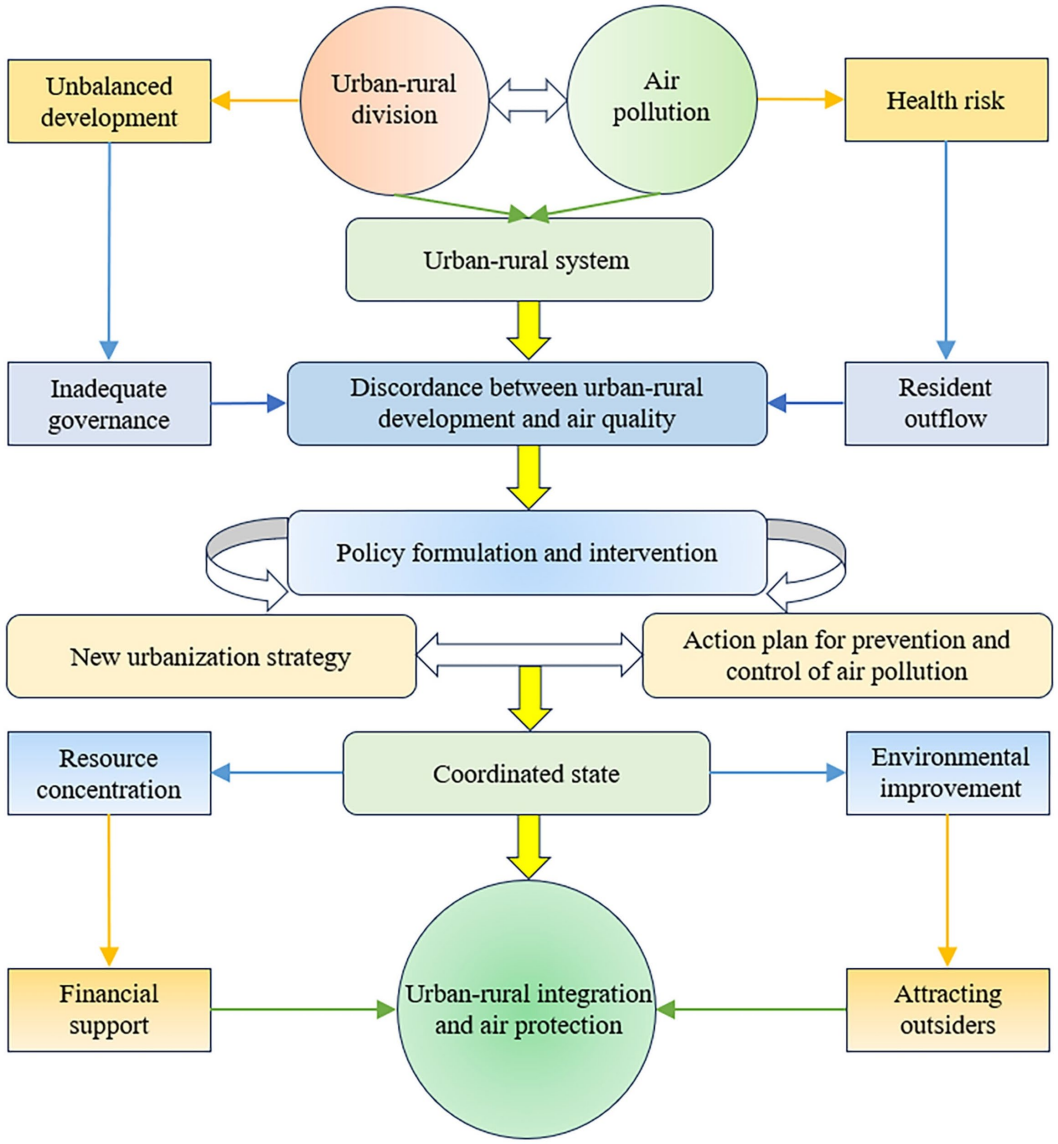
Coupling relationship between urban–rural integration and air quality.

The interaction between urban–rural integration and air quality is mainly manifested in the mutual promotion and mutual constraints of urban–rural synergistic development and atmospheric environmental protection, and only through the realization of the coordinated operation can we guarantee the sustainable development of urban and rural areas (Figure 1). First, the effect of urban–rural integration on air quality. On the one hand, urban–rural integration promotes the flow of urban and rural factors, enabling the concentration of human and physical capital and contributing to urban and rural prosperity and development, but behind the development there may also be the pain of polluting emissions and environmental damage, leading to the deterioration of air quality (42). On the other hand, urban–rural integration promotes the extension of urban infrastructure to the countryside and the coverage of public services and social undertakings to the countryside, which can improve the efficiency of the utilization of factors of production such as capital, labor and energy in urban and rural areas, thus reducing pollution emissions and improving air quality (41). In addition, the strategy of urban–rural integration emphasizes the importance of environmental protection, and to a certain extent, the relevant departments will also give corresponding policies and financial support to increase the strength of environmental protection, thus contributing to the treatment of air pollution and the improvement of air quality.

Second, the effect of air quality on urban–rural integration. On the one hand, good air quality has a catalytic effect on urban–rural integration. With the transformation of human civilization from industrial civilization to ecological civilization, people have higher requirements for the living environment, and good air quality can create a new name card for the city, attract more foreigners to settle down and contribute to the development of the green economy (38). In other words, good air quality can indirectly enhance the core competitiveness of cities and further promote economic urbanization and population urbanization, thereby accelerating urban–rural integration. On the other hand, poor air quality not only leads to climate deterioration, but may also damage the image of the city or even harm human health, restricting the development of the urban economy (56). This leads to outward migration and constrains quality development in urban and rural areas, and can hinder the process of urban–rural integration. Therefore, urban–rural integration and air quality both affect and interact with each other, and shortcomings in either system can hinder overall high-quality development. This paper argues that the maximization of the overall effect of the composite system can only be achieved by finding ways to promote the simultaneous development of urban–rural integration and air quality improvement.

## 3 Methods and data

### 3.1 Indicator system

#### 3.1.1 Level of urban–rural integration development

Establishing a rational indicator system is a prerequisite for measuring the level of urban–rural integration. Specifically, economics emphasizes the rational allocation of production factors between urban and rural areas, sociology asserts that urban and rural residents should have equitable access to social services, ecology highlights the balanced development of urban construction and environmental protection, and geography constructs urban–rural networks based on spatial concepts. Ultimately, urban–rural integration is grounded in a “people-centered” approach, reflecting its multidimensional nature and the convergence of various fields. Following the existing literature (7, 57), this study selected 22 indicators from five dimensions to measure the level of urban–rural integration in Yangtze River middle reaches city cluster (Table 1). Principal component analysis was employed to calculate the level of urban–rural integration development. The formulae are as follows:


(1) $$\left\{ \begin{array}{c} \begin{array}{l} N_{ij}=\frac{M_{ij}-\min\left(M_{ij}\right)}{\max\left(M_{ij}\right)-\min\left(M_{ij}\right)},\mathrm{The}\;\mathrm{indicator}\;\mathrm{attribute}\;\mathrm{is}\;\\ \mathrm{positive} \end{array}\\ \begin{array}{l} N_{ij}=\frac{\max\left(M_{ij}\right)-M_{ij}}{\max\left(M_{ij}\right)-\min\left(M_{ij}\right)},\mathrm{The}\;\mathrm{indicator}\;\mathrm{attribute}\;\mathrm{is}\;\\ \mathrm{negative} \end{array} \end{array}\right.$$

(2) $$URI_{k}=\overset{22}{\underset{i=1}{\sum }}\lambda_{ki}N_{ij}$$

(3) $$URI=\frac{\sum_{i=1}^{k}a_{k}URI_{k}}{\sum_{i=1}^{k}a_{k}}$$

where *M_ij_* represents the original value of the indicator; $\max\left(M_{ij}\right)$ and $\min\left(M_{ij}\right)$ denote the maximum and minimum values of the original values, respectively; and *N_ij_* is the standardized value. $URI_{k}$ represents the level of urban–rural integration development in the *k*-th dimension; $a_{k}$ denotes the variance contribution rate; and *URI* indicates the overall level of urban–rural integration. The study first employed Equation 1 for data standardization, followed by the Kaiser–Meyer–Olkin and Bartlett’s tests of sphericity to verify the suitability of the indicators for principal component analysis. Subsequently, Equation 2 was used to calculate the level of urban–rural integration development in the *k*-th dimension. Finally, Equation 3 was applied to obtain the weighted overall level of urban–rural integration:

**Table 1:** Indicator system for the level of urban–rural integration development.

Dimension	Indicator	Calculation method	Weight (%)	Attribute
Population integration	Employment contrast coefficient	The disparity in urban–rural employment ratio (%)	3.56	Negative
	Population urbanization level	Urban population/total population (%)	5.56	Positive
	Non-agricultural to agricultural employment ratio	Number of employees in the secondary and tertiary industry/number of employees in the primary industry (%)	5.82	Positive
Economic integration	Binary contrast coefficient	(Output value of primary industry/number of employees in the primary industry)/(output value of the secondary and tertiary industry/number of employees in the secondary and tertiary industries) (%)	5.07	Positive
	Comparison of urban- rural residents’ incomes	Per capita disposable income of urban residents/per capita disposable income of rural residents (%)	4.52	Negative
	Comparison of urban–rural residents’ consumption	Per capita consumption expenditure of urban residents/per capita consumption expenditure of rural residents (%)	5.29	Negative
	Urban–rural Engel coefficient ratio	Urban Engel coefficient/rural Engel coefficient (%)	3.45	Positive
	Per capita GDP	Regional GDP/total population (Yuan)	4.83	Positive
Social integration	Comparison of culture, education, and entertainment in urban–rural regions	Per capita expenditure on culture, education, and entertainment for urban residents/per capita expenditure on culture, education, and entertainment for rural residents (%)	4.59	Negative
	Comparison of urban–rural transportation and communication	Per capita transportation and communication expenditure of urban residents/per capita transportation and communication expenditure of rural residents (%)	4.53	Negative
	Comparison of urban–rural health care	Per capita healthcare expenditure of urban residents/per capita healthcare expenditure of rural residents (%)	3.05	Negative
	Coverage of urban- rural pension insurance	Number of urban–rural residents participating in pension insurance/total population (%)	3.89	Positive
	Coverage of urban- rural unemployment insurance	Number of urban–rural residents participating in unemployment insurance/total population (%)	5.63	Positive
Spatial integration	Urban–rural spatial expansion	Agricultural cultivated area/urban built-up area (%)	4.99	Positive
	Land urbanization level	Urban built-up area/total land area (%)	5.52	Positive
	Urban–rural population density ratio	Urban population density/rural population density (%)	4.93	Negative
	Urban–rural transportation network density	Highway operating mileage/total land area (km/km^2^)	4.94	Positive
	Urban–rural spatial circulation medium	Private car ownership/total population (Vehicle/person)	3.98	Positive
Ecological integration	Urban–rural greening level	Green coverage rate (%)	3.93	Positive
	Urban–rural energy conservation and emission reduction	Total energy consumption /GDP (Ten thousand tons of standard coal/ten thousand yuan)	3.88	Negative
	Urban–rural pollution control	Sewage treatment rate (%)	4.47	Positive
	Urban–rural household garbage disposal	Harmless treatment rate of household garbage (%)	3.57	Positive

#### 3.1.2 Level of air quality development

The Air Quality Index (AQI) is a dimensionless relative numerical value quantitatively describing air quality levels (34, 36). A larger AQI indicates more air pollution and poorer air quality. Based on the magnitude of AQI values, air quality was categorized into six levels: 0–50 is excellent, 51–100 is good, 101–150 is mild pollution, 151–200 is moderate pollution, 201–300 is heavy pollution, and > 300 is severe pollution. This study calculated the annual AQI of cities by averaging the monthly AQI values. Because the principal component scores of the urban–rural integration development level had a positive effect on the evaluation of urban–rural integration, higher scores indicated higher levels, whereas higher AQI values signified poorer air quality. To ensure a positive effect on air quality evaluation, the ratio of urban GDP to AQI was utilized to represent the level of air quality development, with higher scores denoting higher levels. Standardization was applied for the coupling analysis. The formula for the calculation is as shown in Equation 4.


(4) $$AQDI_{\mathrm{it}}=GDP_{\mathrm{it}}/\frac{\sum_{j=1}^{12}AQI_{ij}}{12}$$

where *AQDI* represents the level of air quality development; *i* refers to the region; *t* represents the year; and *j* represents the month.

### 3.2 Data sources

**Figure 2:**
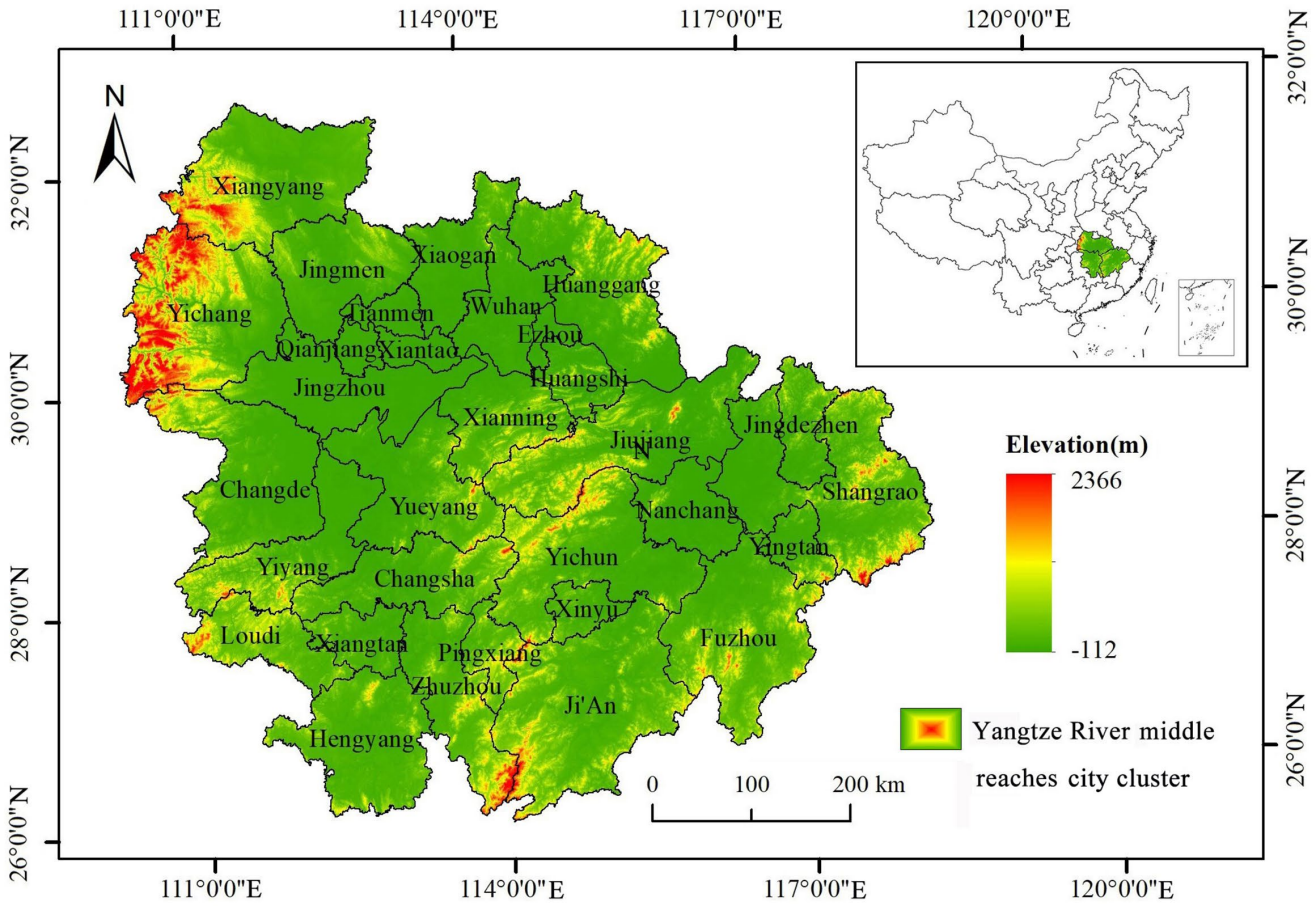
Location of the study area in China.

City clusters have played a pivotal role in China’s economic growth, with a development pattern centered on city cluster rapidly emerging (42). Yangtze River middle reaches city cluster accounts for approximately 10% of China’s population and GDP, underscoring its significant position in the nation’s socio-economic landscape. The status of its development has garnered considerable attention from government authorities. *The 14th Five-Year Plan for the Development of Yangtze River middle reaches city cluster* explicitly emphasizes the need to accelerate regional coordinated development and mitigate severe pollution, with urban–rural integration and air quality improvement as primary objectives. Therefore, investigating urban–rural integration and air quality issues within the context of Yangtze River middle reaches city cluster under this policy guidance is of practical significance and can inform government decision-making. Given the absence of data from Tianmen, Qianjiang, and Xiantao, this study selected the remaining 28 prefecture-level cities in Yangtze River middle reaches city cluster as research subjects (Figure 2). Data for the measurement indicator system of urban–rural integration development were mainly derived from annual statistical yearbooks, statistical bulletins, and government websites. AQI data were obtained from the *Air Quality Online Monitoring and Analysis Platform*.^1^ Considering the accessibility of urban–rural integration and air quality data, this study focused on 2013 to 2021. Data with different statistical calibers were converted accordingly, and missing data were supplemented using linear interpolation.

### 3.3 Methods

#### 3.3.1 Coupling coordination degree model

The coupling coordination degree model is commonly used in studies examining the relationship between urbanization and air quality (41, 52), providing methodological insights for this research. This model quantifies the intensity of interactions between different systems, yielding results that can be categorized into coupling degree and coordination degree. The coupling degree refers to the correlation between systems but does not indicate whether this relationship is positive or negative. A higher coupling degree suggests a closer relationship between the two. In contrast, the coordination degree reflects the quality of their interactions. A higher coordination degree indicates a state of harmony where the systems mutually reinforce each other, resulting in positive outcomes; conversely, a lower coordination degree indicates a state of imbalance where the systems constrain one another, leading to negative effects. This study constructs a coupling coordination degree model to analyze the relationship between the urban–rural integration development level and air quality development level. The formulae are as shown in Equations (5, 6).


(5) $$C=2\sqrt{X_{1}X_{2}}/\left(X_{1}+X_{2}\right)$$

(6) $$D=\sqrt{C\times T};T=\alpha X_{1}+\beta X_{2}$$

where *D* represents the coordination degree between the urban–rural integration development level and air quality development level. *C* represents the coupling degree and *T* represents the comprehensive coordination index. *X_1_* denotes the urban–rural integration system measured by the level of urban–rural integration development. *X_2_* signifies the air quality system measured by the level of air quality development. α and β are the contribution coefficients of urban–rural integration and air quality, respectively. Considering the equal importance of urban–rural integration and air quality to social development, α and β were set to 0.5. This study categorizes the coupling degree and coordination degree into different classifications (Table 2).

**Table 2:** The classification of coupling coordination degree.

Name	Scope	Classification
Coupling degree	0.0	No coupling relationship
	(0.0, 0.3)	Low-level coupling
	(0.3, 0.5)	Antagonism
	(0.5, 0.8)	Adaptation
	(0.8, 1.0)	High-level coupling
	1.0	Benign resonance coupling
Coordination degree	(0.0, 0.1)	Extremely uncoordinated
	(0.1, 0.2)	Seriously uncoordinated
	(0.2, 0.3)	Moderately uncoordinated
	(0.3, 0.4)	Slightly uncoordinated
	(0.4, 0.5)	Nearly uncoordinated
	(0.5, 0.6)	Barely coordinated
	(0.6, 0.7)	Basically coordinated
	(0.7, 0.8)	Moderately coordinated
	(0.8, 0.9)	Favorably coordinated
	(0.9, 1.0)	Superiorly coordinated

#### 3.3.2 Trend surface analysis

Trend surface analysis can be used to analyze trends in discrete spatial data and elucidate the spatial variation and distribution patterns of geographic elements (58). In this study, the coordination degree between urban–rural integration development and air quality development was taken as the observed value. The trend surface method was employed to analyze spatial differentiation characteristics. Z*i* (x*_i_*,y*_i_*) represents the actual observed value of the *i*-th geographic element, and T*i* (x*_i_*,y*_i_*) denotes the fitted value of the trend surface. The formula is as shown in Equation 7.


(7) $$Z_{i}\;\left(x_{i},y_{i}\right)=T_{i}\;\left(x_{i},y_{i}\right)+\varepsilon_{i}$$

where (x*_i_*, y*_i_*) denotes the planar spatial coordinates and $\varepsilon_{i}$ represents the residual, namely the deviation between the actual and fitted values.

#### 3.3.3 Geographic detector

Geographic detectors are widely employed to assess the interactions among multiple influencing factors (59). When the dependent variable *Y* and the independent variable *X* are numerical, discretizing *X* into categorical variables and utilizing the relationship established by the geographic detector yields more reliable results than classical regression, particularly when the sample is <30. Conversely, the geographic detector assumes nonlinearity in the variables, falling within the domain of analysis of variance, indicating how much explanatory power the independent variable *X* has on the dependent variable *Y*. In this study, a geographic detector was employed to explore the relationship between the coordination degree *Y* of urban–rural integration development and air quality development, and the driving factor *X*. The model is expressed as shown in Equation 8.


(8) $$q=1-\frac{1}{N\sigma^{2}}\overset{m}{\underset{h=1}{\sum }}N_{h}\sigma_{h}^{2}$$

where *q* is the detection value of influencing factor *X*; *m* represents the stratification of driving factors; *N* and *N_h_* are the sample sizes of the study area and the detection area, respectively; and $\sigma^{2}$ and $\sigma_{h}^{2}$ are the variances of the *Y* values in the study area and the detection area, respectively. This study considers the internal factors constituting urban–rural integration and air quality systems as driving factors *X* and converts them into categorical variables. Additionally, considering that terrain and infrastructure conditions may affect the coordination degree, factors such as terrain ruggedness and fixed asset investments were included as driving factors.

## 4 Results

### 4.1 Analysis of urban–rural integration development and air quality development

#### 4.1.1 Analysis of urban–rural integration development

This study measures the level of urban–rural integration development in different cities, and then uses the average value to measure the level of urban–rural integration development in Yangtze River middle reaches city cluster (Table 3). Overall, the urban–rural integration development level in Yangtze River middle reaches city cluster showed a steady upward trend, indicating the region’s emphasis on urban–rural development and its positive outcomes. The level of urban–rural integration development increased from 0.394 in 2013 to 0.507 in 2021. The evolutionary trend can be divided into three stages: from 2013 to 2015, the level of urban–rural integration development increased from 0.394 to 0.423, indicating a slow increase. In 2015–2016, the level of urban–rural integration development in some cities declined slightly, which is related to the context of supply-side structural reforms undertaken in China and the shift to a higher-quality concept of urban–rural integration. From 2017 to 2021, the level of integrated urban–rural development increased from 0.423 to 0.507, showing a rapid growth trend. From the results of the sub-dimensions, we measured the level of urban–rural population integration, urban–rural economic integration, urban–rural social integration, urban–rural spatial integration, and urban–rural ecological integration of city cluster according to the weights of the indicators. Taking 2021 as an example, the five dimensions were calculated as 0.076, 0.117, 0.110, 0.124 and 0.080, respectively. It can be seen that the integration of spatial and economic elements in the process of urban–rural integration is relatively better, and the integration of population and ecological elements is relatively weak. Therefore, the future urban–rural development in Yangtze River middle reaches city cluster should pay more attention to population mobility and environmental protection in order to maximize the overall benefits.

**Table 3:** The level of urban–rural integration development during the research period.

Region	2013	2014	2015	2016	2017	2018	2019	2020	2021
Wuhan	0.523	0.553	0.555	0.565	0.591	0.609	0.638	0.628	0.637
Huangshi	0.385	0.468	0.457	0.438	0.472	0.466	0.473	0.489	0.504
Yichang	0.427	0.449	0.459	0.460	0.487	0.487	0.486	0.524	0.541
Xiangyang	0.404	0.441	0.434	0.460	0.470	0.466	0.476	0.491	0.526
Ezhou	0.576	0.579	0.557	0.530	0.539	0.567	0.528	0.548	0.549
Jingmen	0.391	0.398	0.376	0.393	0.413	0.426	0.436	0.449	0.495
Xiaogan	0.375	0.384	0.386	0.399	0.425	0.432	0.438	0.406	0.458
Jingzhou	0.336	0.356	0.364	0.402	0.436	0.445	0.457	0.473	0.560
Huanggang	0.232	0.325	0.310	0.347	0.354	0.331	0.347	0.413	0.446
Xianning	0.381	0.428	0.428	0.402	0.440	0.436	0.436	0.455	0.490
Changsha	0.552	0.556	0.581	0.579	0.600	0.612	0.630	0.597	0.612
Zhuzhou	0.396	0.443	0.473	0.469	0.470	0.475	0.507	0.489	0.519
Xiangtan	0.565	0.461	0.483	0.471	0.596	0.591	0.597	0.506	0.525
Hengyang	0.407	0.420	0.444	0.427	0.443	0.456	0.479	0.494	0.499
Yueyang	0.388	0.433	0.398	0.391	0.424	0.440	0.451	0.484	0.502
Changde	0.417	0.425	0.448	0.453	0.463	0.457	0.468	0.483	0.498
Yiyang	0.397	0.427	0.423	0.424	0.434	0.449	0.455	0.480	0.491
Loudi	0.344	0.351	0.347	0.381	0.415	0.441	0.502	0.444	0.467
Nanchang	0.402	0.429	0.434	0.442	0.474	0.487	0.499	0.519	0.546
Jingdezhen	0.330	0.396	0.413	0.395	0.407	0.446	0.482	0.470	0.483
Pingxiang	0.367	0.392	0.417	0.425	0.442	0.434	0.481	0.512	0.510
Jiujiang	0.362	0.362	0.371	0.383	0.399	0.413	0.434	0.458	0.474
Xinyu	0.436	0.471	0.473	0.433	0.476	0.466	0.477	0.516	0.519
Yingtan	0.348	0.444	0.423	0.415	0.445	0.415	0.467	0.498	0.499
Ji’an	0.254	0.329	0.332	0.326	0.352	0.357	0.396	0.437	0.455
Yichun	0.374	0.403	0.369	0.360	0.400	0.418	0.458	0.466	0.493
Fuzhou	0.356	0.375	0.356	0.344	0.400	0.399	0.431	0.446	0.460
Shangrao	0.305	0.331	0.332	0.331	0.350	0.358	0.385	0.413	0.440
Average value	0.394	0.422	0.423	0.423	0.451	0.456	0.476	0.485	0.507

Regional disparities are evident in the development of urban–rural integration. High-value areas of urban–rural integration development are primarily concentrated in the Wuhan, Changsha-Zhuzhou-Xiangtan, and Nanchang metropolitan areas, represented by cities such as Wuhan, Changsha, Nanchang, Ezhou, and Xiangtan. Conversely, some cities distant from these major metropolitan areas, such as Shangrao and Changde, exhibited lower levels of urban–rural integration development, constrained by factors such as resources and geographical location. However, the standard deviation changes in urban–rural integration development within Yangtze River middle reaches city cluster from 2013 to 2021 indicated a gradual reduction in regional disparities. This indicates that the development of city cluster is paying more and more attention to the driving effect of the central city on the surrounding areas in order to realize the balanced development of the region as a whole.

#### 4.1.2 Analysis of AQI

Overall, the AQI in Yangtze River middle reaches city cluster showed a significant downward trend, indicating an improvement in air quality. The average AQI decreased from 164.893 in 2013 to 66.759 in 2021, with air quality shifting from moderate pollution to good. This can be roughly divided into two stages. From 2013 to 2016, there was a rapid decline phase, with the AQI dropping from 164.893 to 79.483. The implementation of the “*Action Plan for Prevention and Control of Air Pollution*” in 2013 was crucial in improving air quality, and the new air quality assessment standard has been rigorously monitoring atmospheric conditions, leading to the establishment of a new mechanism for air pollution prevention and control through several policies. The period from 2017 to 2021 saw a fluctuating decline, with the AQI decreasing from 79.850 to 66.750. The “*Three-Year Action Plan to Fight Air Pollution*,” issued in 2018, similarly enhanced public satisfaction. However, because air quality has already reached a relatively high level, it has become more challenging to upgrade from good to excellent air quality.

Regional analysis revealed that, during the study period, the disparity in AQI among various cities decreased, transitioning from an initial disordered state to a more orderly reduction. Wuhan, Changsha, and Jiujiang experienced the most significant reductions in AQI, with their air quality shifting from heavy to moderate pollution to good, indicating notable achievements in air pollution control in these cities. Conversely, in cities such as Xiangyang, Yiyang, and Pingxiang, there was a slight rebound in the AQI, necessitating intensive air pollution control efforts. Jingdezhen was the only city where the air quality transitioned to excellent during the study period. Notably, the spatial distribution characteristics of air quality within city clusters resemble those of urban–rural integration, as evidenced by larger cities such as Wuhan and Changsha exhibiting higher levels of urban–rural integration and better air quality (Table 4).

**Table 4:** The AQI during the research period.

Region	2013	2014	2015	2016	2017	2018	2019	2020	2021
Wuhan	225.000	112.833	103.417	92.500	89.250	78.667	88.000	72.167	73.750
Huangshi	154.476	96.000	93.830	85.000	82.580	72.420	79.920	67.920	65.500
Yichang	138.000	123.083	98.000	91.750	90.417	78.750	91.167	71.000	77.250
Xiangyang	166.547	91.579	111.083	101.333	100.917	87.083	99.750	85.917	94.250
Ezhou	172.267	84.000	100.670	93.170	84.170	75.170	80.330	70.420	67.250
Jingmen	167.558	114.000	101.500	87.920	83.080	83.670	95.500	77.920	84.750
Xiaogan	167.690	91.000	100.417	80.417	83.917	76.250	83.167	67.500	69.750
Jingzhou	169.000	119.920	101.830	92.920	86.330	77.330	82.500	68.580	72.500
Huanggang	174.293	82.000	89.830	83.750	84.970	76.170	80.670	68.750	61.500
Xianning	159.184	117.000	87.167	79.167	78.833	67.083	76.667	61.167	60.500
Changsha	177.000	100.917	86.833	82.750	85.083	76.250	83.417	70.667	75.500
Zhuzhou	172.000	97.917	80.583	80.083	82.167	69.917	80.000	66.333	73.750
Xiangtan	168.000	97.583	82.667	78.167	82.250	75.417	82.500	67.083	76.250
Hengyang	169.788	110.000	79.170	77.970	76.330	64.500	71.670	61.920	63.000
Yueyang	125.000	90.750	80.667	78.750	76.417	73.667	78.167	65.083	64.500
Changde	117.000	93.080	77.830	82.000	83.580	69.160	81.580	68.330	71.750
Yiyang	166.556	119.000	81.250	76.167	74.917	65.500	86.833	69.667	77.000
Loudi	167.304	109.000	78.500	71.583	69.250	61.500	68.500	56.833	63.000
Nanchang	135.000	74.917	68.750	73.667	75.583	63.250	70.583	65.083	64.750
Jingdezhen	160.432	56.000	63.000	65.500	65.170	52.830	53.833	48.083	48.250
Pingxiang	154.818	83.000	78.833	82.583	78.667	65.583	70.833	60.750	67.000
Jiujiang	229.000	68.833	74.417	79.333	79.917	71.667	79.417	68.500	64.500
Xinyu	167.751	52.000	60.750	67.167	74.250	61.833	64.667	56.333	56.750
Yingtan	162.758	95.000	66.167	68.667	70.333	63.833	67.083	60.833	53.500
Ji’an	168.266	53.000	59.670	66.580	79.420	65.250	65.920	59.330	56.250
Yichun	155.746	124.000	58.833	74.917	75.667	62.583	66.250	56.333	57.250
Fuzhou	162.101	74.000	62.420	63.250	70.920	60.420	61.830	55.080	53.750
Shangrao	164.485	89.000	63.917	68.750	71.417	60.417	62.250	56.583	55.500
Average value	164.893	93.550	81.857	79.493	79.850	69.863	76.893	65.149	66.759

### 4.2 Spatiotemporal evolution of the coordination degree

#### 4.2.1 Temporal evolution of the coordination degree

**Figure 3:**
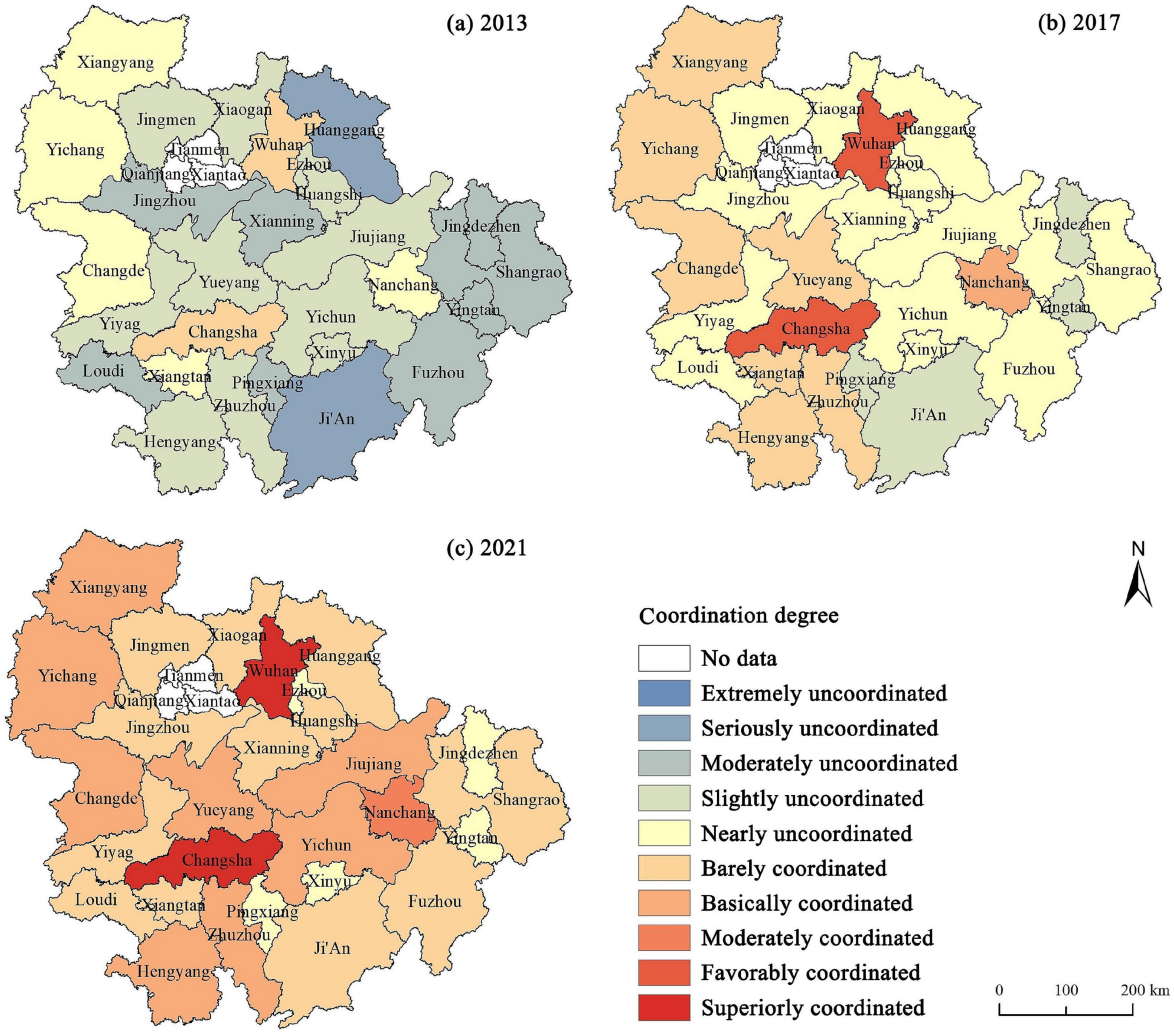
The changes in the coordination degree between urban–rural integration and air quality in 2013, 2017 and 2021.

This study presents the change in the coordination degree between urban–rural integration and air quality (Figure 3). Overall, from 2013 to 2021, the degree of coupling and coordination between urban–rural integration and air quality development in Yangtze River middle reaches city cluster showed a steady upward trend. The coupling degree increased from 0.570 in 2013 to 0.794 in 2021, and the coordination degree increased from 0.337 in 2013 to 0.591 in 2021. The coupling type has transitioned from a phase of adaptation to approaching high-level coupling, indicating an enhanced interaction between urban–rural integration and air quality, deepening their mutual influence. The coordination type shifted from slightly uncoordinated to barely coordinated, indicating that the relationship between urban–rural integration and air quality shifted from mutually constraining to mutually reinforcing. This result reflects the strengthening connection between urban–rural integration and air quality over time, with their mutual reinforcement contributing to the environmentally friendly development of city cluster. However, the overall level of coordination degree is not high, and there is a large gap between cities, indicating that the task of air pollution prevention and control in the process of urban–rural integration is still arduous. Additionally, the standard deviation of the coordination degree increased from 0.102 in 2013 to 0.123 in 2021, indicating a widening regional disparity in the coupling coordination between urban–rural integration and air quality.

Regarding regional distribution, regions with high coordination degree were mainly concentrated in Wuhan, Changsha, Nanchang, and Yichang, with Wuhan having the highest coordination degree in 2021. However, the number of cities within city clusters that achieve high-quality coordination remains limited, indicating substantial potential for improvement in inter-city coordination. Regions with lower coordination levels are predominantly located on the periphery of the city cluster, including cities such as Pingxiang, Ji’an, and Yingtan, which are manifested as small and medium-sized cities with a weak economic base. Furthermore, the degree of interaction matching between urban–rural integration and air quality has deepened in most cities, with cities of coordinated types gradually increasing. From this, it can be inferred that as the concept of green development has received increasing attention, the relationship between urban–rural integration and air quality in Yangtze River middle reaches city cluster will become more coordinated in the future. However, certain small and medium-sized cities on the brink of an imbalance require further measures.

#### 4.2.2 Spatial evolution of the coordination degree

**Figure 4:**
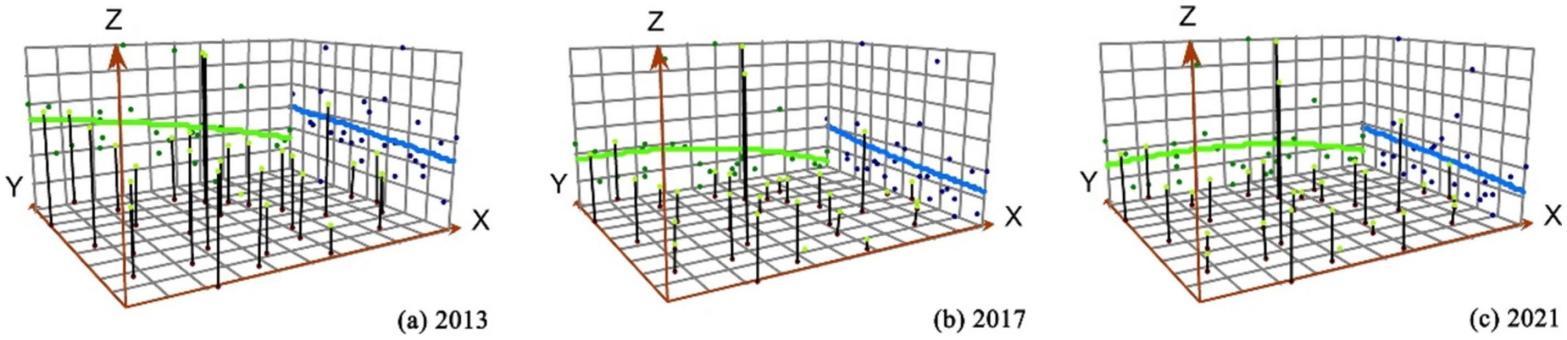
Trend surface changes of coordination degree in 2013, 2017 and 2021.

This study initially calculates Moran’s I of the coordination degree of the historical levels of urban–rural integration and air quality development. The majority of the results did not pass the significance test, indicating an overall dispersed spatial distribution of coordination degree. Subsequently, the spatial evolution of coordination degree was investigated using trend surface analysis (Figure 4). The X-axis represents the eastern direction, and the Y-axis represents the northern direction. The green curve represents the fitted line of change in the east–west direction, whereas the blue curve represents the fitted line of change in the north–south direction. The figure shows that the trend lines of the coordination degree exhibit a distribution feature of “high in the west and low in the east, high in the north, and low in the south.” The trend lines in the east–west direction were steeper than those in the north–south direction, indicating a more pronounced differentiation of coordination degree in the east–west direction. Meanwhile, the trend lines in the east–west direction show a tendency towards gradual flattening, indicating a diminishing gap in the coordination degree along this axis. Conversely, no significant change was evident in the trend lines in the north–south direction. Furthermore, the spatial distribution of coordination degree exhibited a three-core structure, with Wuhan, Changsha, and Nanchang at the center.

### 4.3 Driving factors of the coordination degree

This study references the findings of Zhang et al. and Li et al. to identify 22 internal factors (Table 1) from the urban–rural integration and air quality systems as potential influences on coordination levels (60, 61). The geographic detector model was utilized to screen all factors based on their q-values (62). Ultimately, the top five factors with the highest q-values were retained as drivers of the coupling coordination between urban–rural integration and air quality. This study found that per capita GDP, the non-agricultural employment ratio, urban–rural spatial circulation media, population urbanization level, and fixed asset investment had relatively higher q-values (Table 5). Therefore, these five factors are the core driving factors of the coupling coordination between urban–rural integration and air quality in Yangtze River middle reaches city cluster. Notably, the q-value only reflects the magnitude of the driving force of each factor on coupling coordination, with a higher q-value indicating a greater driving effect but without implying a linear relationship. The subsequent sections attempt to analyze the driving mechanisms.

**Table 5:** Geographic detection results of driving factors for coordination degree.

Year	Per capita GDP	Non-agricultural employment ratio	Urban–rural spatial circulation medium	Population urbanization level	Fixed asset investment
2013	0.520^**^	0.488^**^	0.388^*^	0.258	0.606^**^
2014	0.423^*^	0.299	0.263	0.406^*^	0.671^***^
2015	0.387^*^	0.223	0.311	0.385	0.673^**^
2016	0.366	0.172	0.371	0.314	0.711
2017	0.460^**^	0.409^*^	0.397^*^	0.282	0.674^***^
2018	0.309	0.446^*^	0.418^*^	0.297	0.675^**^
2019	0.448^**^	0.149	0.338	0.442^**^	0.685^***^
2020	0.172	0.142	0.130	0.419^*^	0.675^***^
2021	0.428^*^	0.233	0.168	0.407^*^	0.685^***^

***, **, and * indicate significance at the 1, 5, and 10% levels, respectively.

Per capita GDP. Per capita GDP reflects the economic development level of a region, providing better economic support for improving living standards, infrastructure construction, ecological environment governance, and the popularization of public services. With the improvement in regional economic strength, the ability of urban areas to support rural areas is also enhanced, and various resources begin to spread to rural areas, promoting urban–rural integration. In addition, the economic development of urban and rural areas is shifting from rapid to high-quality, with the proportion of economic investment in the restoration and governance of urban and rural ecological environments continuously increasing, effectively improving air quality. Therefore, an increase in per capita GDP contributes to enhancing the coordination degree between urban–rural integration and air quality.

Non-agricultural employment ratio. The non-agricultural employment ratio reflects the development status of urban–rural industries. Generally, an increase in non-agricultural employment implies the development of non-agricultural industries, optimizing the industrial structure of the region and gradually entering a stage of industrialization and modernization with low input and high returns. Simultaneously, the development of non-agricultural industries promotes the prosperity of rural industries and increases residents’ income, which is conducive to narrowing the urban–rural gap and promoting urban–rural integration. Furthermore, the development of non-agricultural industries, such as the emergence of a batch of high-tech industries, can reduce air pollution through technological progress. Therefore, promoting the non-agricultural employment ratio is conducive to enhancing the coordination degree between urban–rural integration and air quality.

Urban–rural spatial circulation medium. The urban–rural spatial circulation medium is a major factor reflecting the convenience of transportation and determining the travel route of urban–rural residents. The development of urban–rural spatial circulation media makes travel more convenient in urban–rural areas, and the flow of factors is more convenient in urban–rural areas, thus promoting urban–rural integration. However, the increase in private car ownership also exacerbates air pollution. Automobile exhaust is the main source of air pollution, which adversely affects air quality. Therefore, an increase in urban–rural spatial circulation media can exacerbate the gap between urban–rural integration and air quality, leading to a decrease in coordination degree.

Population urbanization level. An increase in the population urbanization level indicates that the population flows from rural to urban areas, which are prone to aggregation at the junction of urban–rural areas. Population aggregation promotes economic output and stimulates consumption, generating more active human activities and promoting urban–rural integration. However, areas with higher urbanization levels may have higher requirements for social services and environmental standards, emphasizing ecological environmental quality. Therefore, an increase in the population urbanization level will promote coordination degree between urban–rural integration and air quality.

Fixed asset investment. The increase in fixed asset investment enables the continuous input of production factors such as resources, funds, technology, and manpower into urban and rural areas, injecting lasting momentum into the economic and social development of urban and rural areas and directly driving the development of urban–rural integration. Simultaneously, with an increase in fixed asset investment, the government also has the ability to regulate air pollution. Therefore, an increase in fixed asset investment promotes coordination degree between urban–rural integration and air quality.

## 5 Discussion

### 5.1 Comparison of related studies

The results of this study confirm that urban–rural integration and air quality may promote each other to achieve coordinated development, or hinder each other to lead to imbalance, consistent with the results of Li et al. (42). Additionally, this research identifies the influences of factors such as economic growth, industrial structure, and transportation conditions on the coordinated development of urban–rural integration and air quality, paralleling the conclusions of Liu et al. (52). Thus, through empirical analysis and comparison with existing literature, the results of this study are substantiated and may provide valuable insights for other regions facing similar challenges.

However, existing literature primarily focuses on the relationship between urbanization and air quality (4, 45), lacking analysis of elements related to urban–rural integration, such as small towns and new rural communities. Currently, while urban–rural integration in China contributes to regional economic growth and social development (63), large-scale township construction poses threats to the ecological environment, with increased pollution emissions adversely affecting air quality and public health (6, 53). Therefore, promoting coordinated development between urban–rural integration and air quality is crucial for achieving long-term sustainable development in these areas. This study employs a coupling coordination degree model to explore their interactions and the factors influencing their coordination. A key theoretical contribution of this work is the integration of urban–rural integration and air quality within a unified analytical framework, examining their feedback mechanisms based on the environmental Kuznets curve theory and systems theory, thereby providing theoretical guidance for long-term sustainable urban–rural development.

Building on this foundation, the study measured the levels of urban–rural integration, air quality, and their coupling coordination degree across different cities. The findings indicate significant variations in urban–rural integration and air quality among cities, with many regions experiencing a misalignment that necessitates targeted remedial actions. However, existing literature lacks direct studies linking urban–rural integration to air quality, providing no basis for coordinated development or air quality improvement. Thus, a practical contribution of this paper is the assessment of the relationship between urban–rural integration and air quality using appropriate indicators and detailed data, offering valuable data references and practical guidance for policymakers in formulating urban development strategies tailored to different cities.

### 5.2 Policy implications

The research results show that the urban–rural integration and air quality of Yangtze River middle reaches city cluster are gradually changing in the direction of high-quality coordination. However, there are great differences among cities, regional imbalance is prominent, and coordination degree is driven by multidimensional factors. Based on these findings, the following recommendations are proposed:

1. This study focuses on the urban–rural integration and air quality status of uncoordinated cities, leveraging the driving factors of their coupling coordination to promote both integration and air quality improvement. On the one hand, urban–rural integration and air quality coupling in most cities are in a state of barely coordinated, and some cities even show no coordination. In order to reverse the uncoordinated state, it is essential to transform development outcomes into enhancements in ecological quality, such as implementing economic strategies that directly address air pollution control, thereby shifting from a traditional model of “pollute first, treat later” to a green, pollution-free development approach. Guiding cities from uncoordinated to coordinated will be crucial for advancing sustainable urban–rural development in the future. On the other hand, results from geographical detectors highlight the importance of factors such as economic development, industrial structure, and transportation conditions. To harness these factors effectively, priority should be given to supporting the development of high-tech industries, achieving green GDP growth and optimizing industrial structures, promoting urban–rural transportation integration, reducing private car usage, and enhancing public transport coverage, all aimed at achieving coordinated development between urban–rural integration and air quality.
2. Strengthening regional cooperative relationships to establish an inclusive and balanced Yangtze River middle reaches city cluster. The overall coordination degree of the study area is insufficient, indicating that urban–rural integration and air quality of coordinated development in city cluster need to be strengthened. From the perspective of urban–rural integration, it is essential to promote healthy competition among cities within the city cluster, dismantling policy and geographical barriers while fostering collaborative development among the three provinces. This approach should aim to create a complementary advantage model among various city cluster and prefecture-level cities. Concurrently, support for inter-provincial and inter-departmental governance should be strengthened, fully leveraging the resource endowments and economic development advantages of each province. This will facilitate the optimization, adjustment, and upgrading of industrial structures, jointly developing industry clusters that capitalize on the advantages of the Yangtze River middle reaches city cluster, thereby enhancing overall coordination. In terms of improving air quality, it is crucial to promote the interconnectedness of air quality information across the city cluster, establishing a shared platform for air pollution exceedance alerts to enable timely responses to pollution control measures. Moreover, while pursuing higher economic benefits, the city cluster must reinforce the constraints of green and pollution-free development in its planning, ensuring a sustained commitment to a path of green development.
3. Based on three indexes, namely, the level of urban–rural integration, the level of air quality and the coordination degree, different categories of cities are classified and differentiated development paths are adopted according to local conditions. The coupled relationship between urban–rural integration and air quality in different city can be categorized into the following four categories: the first category consists of fast-growing, low-polluting cities, represented by Wuhan and Changsha, among others. These cities have a high starting point for development and strong resource allocation efficiency, have spawned a number of high-tech industries, and have promoted rapid urban–rural development while focusing on environmental management, with a high coordination degree. The second category is the fast-developing and highly polluting cities, represented by Xiangyang and Jingmen. Although these cities have a high level of urban–rural integration development, their air quality is poor, and their coupling relationships are mostly on nearly uncoordinated or barely coordinated, indicating that the management of air pollution has been neglected in the process of urban–rural integration and development. The third category is low-pollution cities with slow development, represented by Fuzhou and Shangrao. These cities have good air quality but low levels of urban–rural integration development, indicating that the role of good air quality in promoting urban–rural integration has not been realized. The fourth category is highly polluted cities with slow development, represented by Huanggang and Xiaogan. These cities have a low level of urban–rural integration development, and their air quality is not as good as it could be, with a low coordination degree. For rapidly growing low-pollution cities, it is crucial to leverage their role as exemplars and catalysts by developing resource-sharing metropolitan areas that foster coordinated development in surrounding regions. In rapidly developing high-pollution cities, efforts should focus on transforming urban–rural development models and pursuing green development pathways, with a strong emphasis on air pollution control. For slowly growing low-pollution cities, the priority should be to promote green economies, such as nurturing environmentally sustainable cultural and tourism towns, and facilitating urban–rural integration through industrial and urban development synergies. In the case of slowly growing high-pollution cities, provincial governments should provide policy support and resource allocation, while also advancing urban–rural integration and air pollution management, necessitating robust governmental guidance.

### 5.3 Research limitations and future direction

This study contributes to the promotion of sustainable urban–rural development and air quality improvement, but some limitations of the study should be mentioned. On the one hand, we analyzed the coordinated relationship between urban–rural integration and air quality at the city level, but lacked the content of measurement at the county level, which is where the urban–rural integration strategy is concerned. The reasons for this are, firstly, it is difficult to collect data on urban–rural integration and air quality at the county level, and secondly, the effect of urban–rural integration at the county level has not yet appeared, and the conditions are not yet mature enough to carry out the relevant research. On the other hand, the AQI is simplified into a single numerical form from a variety of pollutants such as PM2.5, PM10, CO, NO2, etc., and the Spatio-temporal evolution of different pollutants in the process of urban–rural integration and the effects they cause have not been clarified. We believe that studying the detailed relationship between different pollutants and urban–rural integration can lead to more specific recommendations in upgrading urban–rural habitat. Therefore, the study of urban–rural integration at the county level and the impact of different pollutants on urban–rural integration development are directions that can be further expanded in future research.

## 6 Conclusion

Exploring the intrinsic relationship between urban–rural integration and air quality in China is vital for reducing air pollution and achieving sustainable urban–rural development. To clarify the interaction between these two factors, this study assesses the levels of urban–rural integration and air quality in Yangtze River middle reaches city cluster from 2013 to 2021, and analyzes their coupling coordination relationship and driving factors. The main conclusions are as follows: (1) The level of urban–rural integration is generally on the rise, with regional disparities that are gradually narrowing. (2) The air quality index shows a marked decline, with air quality classifications improving from moderate pollution to good. (3) The coupling and coordination degrees between urban–rural integration and air quality are steadily increasing, indicating a state of coordinated development over time, with their mutual reinforcement contributing to the environmentally friendly development of city cluster. The spatial distribution of coordination shows a decentralized pattern, characterized by higher values in the west and lower values in the east, as well as higher values in the north and lower values in the south. (4) The key drivers of coordinated development between urban–rural integration and air quality include per capita GDP, the non-agricultural employment ratio, urban–rural spatial circulation media, the level of population urbanization, and fixed asset investment. The findings provide a basis for enhancing urban–rural integration and improving air quality in Yangtze River middle reaches city cluster, and the policy implications may also serve as a reference for similar city cluster.

## Data Availability

Publicly available datasets were analyzed in this study. This data can be found at: https://data.cnki.net/.

## Footnotes

^1^ https://www.aqistudy.cn/
